# A Reference Hand Book of the Medical Sciences

**Published:** 1887-01

**Authors:** 


### BIBLIOGRAPHY.

A REFERENCE HAND BOOK OF THE MEDICAL SCIENCE.—Embracing the entire range of Scientific and Practical Medicine and allied Science; by various writers; illustrated by chromo—lithographs and fine wood engravings. Edited by Albert H. Buck, M. D., New York City; vol. III, New York, 1886. Wm. Wood & Co., 56 and 58 Lafayette Place. Cloth; 812 pages.

This is the 3rd volume of this great work. Some idea may be had of its scope when we reflect that it is to be complete in seven volumes, and this, the third, only brings us down to Fac–Hays—embracing every subject connected with medical and sanitary science between those letters.

It would be tedious to enumerate the subject herein treated of, and perhaps unfair to specify any one, where all are so fully and so ably written upon. The chapter on "The Poisonous and Edible Fungi" particularly attracted our attention, and induced a careful reading; perhaps, from the fact that it is embellished with beautiful and life-like colored lithographs. Everything that is known about every kind of mushroom is here related: How to distinguish the poisonous from the edible; how to prepare those which are used for food; how to propagate them. A description is given of their extensive cultivation for market in Paris, and it is stated that they are grown from seed (?) *Champignon blanc*—white mushroom. The perusal of this chapter will interest any one. The chapter on "Military and Naval Hygiene" alone is a valuable treatise, and contains a vast amount of useful information. Were we to undertake to "review" this work as it deserves, it would occupy more time and space than we can afford to bestow. We will say this: No physician's library can be considered complete without it. It is by far the most important work issued from the medical press since the Surgical History of the War (Rebellion?)

